# Characterization of the complete mitochondrial genome of *Coelastrum_sp.*F187

**DOI:** 10.1080/23802359.2017.1357440

**Published:** 2017-07-26

**Authors:** Zhao Kai Wang, Li Juan He, Fan Hu, Xiang Zhi Lin

**Affiliations:** Engineering Research Center of Marine Biological Resource Comprehensive Utilization, Third Institute of Oceanography, State Oceanic Administration, Xiamen, Fujian, P.R. China

**Keywords:** *Coelastrum* sp., mitochondrial genome, rare genus, green algae, phylogenetic tree

## Abstract

*Coelastrum* is a genus of green algae that belongs to the *Scenedesmaceae* family. There is little information available about this genus. A phylogenetic analysis of the ITS2 sequences showed that *Coelastrum* is a paraphyletic group. To better explore the phylogenetic status of this genus, we report the mitochondrial genome sequence of *Coelastrum* sp. F187 using next-generation sequencing technology. The complete mitochondrial genome is 52,888 bp in size and encodes 43 conventional mitochondrial genes, including 14 protein-coding genes (PCGs), 24 transfer RNA (tRNA) genes, and four ribosomal RNA (rRNA) genes. Most of the PCGs (12/14) and all tRNAs were located in the heavy chain and the light chain, respectively. The phylogenetic analysis based on the complete mitochondrial genome sequences indicated that *Coelastrum* is closely related to *Pectinodesmus pectinatus*. The sequenced complete mitochondrial genome of *Coelastrum* sp. F187 provides fundamental molecular data that will be useful for species identification, population genetics, and evolutionary relationships.

## Introduction

Although numerous mitogenomes have been sequenced in recent years (Cameron 2014), little is known about the mitochondrial genome of *Coelastrum*, a rare genus of green algae belonging to the *Scenedesmaceae* family. In our previous study, we isolated a microalgae morphologically designated to the *Coelastrum* genus (*Coelastrum* sp. F187) from Furong Lake in Fujian, China and deposited in Marine Medicinal Organism Germplasm Resources Bank of Third Institute of Oceanography (MMOGRB 0187F). The ITS analysis results indicated that it was primarily related to the strain *Coelastrum astroideum var. rugosum* UTEX2442 (Dong et al. 2013). To determine the evolutionary and phylogenetic origin of *Coelastrum* sp. F187, its complete mitochondrial genome was sequenced.

The mitochondrial genome of *Coelastrum* sp. F187 was sequenced with the Illumina HiSeq™ 2500 platform at the Beijing Genomic Institute (BGI, Shenzhen, China), by using the PE250 paired-end sequencing strategy. Approximately 3.7 Gb of raw data were generated and finally a total of 14.86 M raw reads were used to construct the genome. All published mitochondrial genome sequences available in NCBI were employed as references for the genome assembly with SOAPdenovo2 (http://soap.genomics.org.cn). Preliminarily gene annotation was conducted using the online program Dual Organellar GenoMe Annotator (DOGMA) (Wyman et al. 2004) and ORF Finder (Cheng et al. 2013) with the *Scenedesmus obliquus* mitochondrial genetic code and default conditions. To verify the exact gene and exon boundaries, the putative gene sequences and protein sequences were BLAST searched in the Nt and Nr databases. All transfer RNA (tRNA) genes were further confirmed using the online web servers tRNAscan-SE and ARWEN (Griffiths-Jones et al. 2003; Schattner et al. 2005; Abe et al. 2011). Phylogenetic analysis was performed using the maximum likelihood analysis implemented in MEGA6 (Tamura et al. 2013).

**Figure 1.**
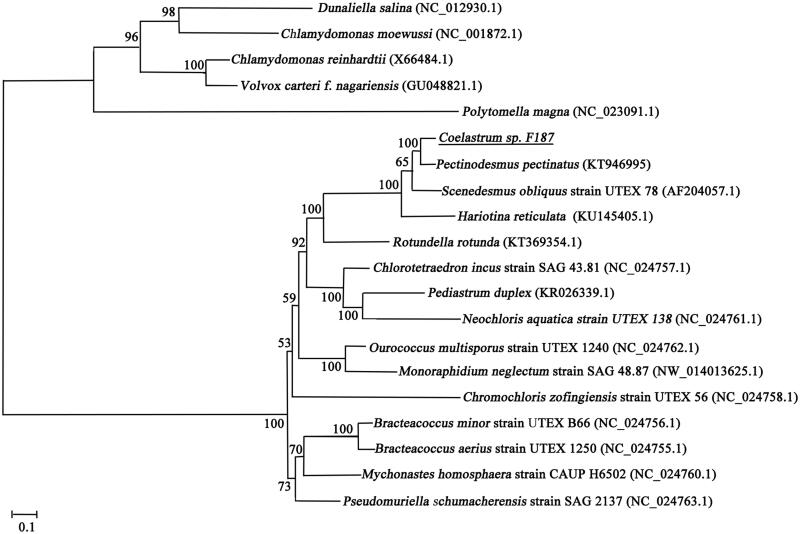
Phylogeny of *Coelastrum* sp. F187 and other *Chlorophyceae* species based on the maximum likelihood (ML) analysis.

The complete mitogenome of *Coelastrum* sp. F187 has a total length of 52,888 bp, and the GC content of the mitochondrial DNA is 40.91%. The annotated genomic sequence can be obtained with accession number KY984063 in GenBank. The genome sequence contains 43 mitochondrial genes, including 14 protein-coding genes (PCGs), 24 tRNA genes, and 4 rRNA genes. The heavy chain encodes 11 PCGs and 1 tRNA gene, whereas the light chain encodes 2 PCGs, most of the tRNA genes (23/24) and all rRNA genes. The base composition of the *Coelastrum* sp. F187 mitochondrial genome is as follows: A (28.2%), T (30.8%), C (18.9%), and G (22.1%), with a total A + T content of 59%. To further explore its taxonomic status, maximum likelihood analyses of *Coelastrum* sp. F187 and 20 other published mitochondrial genomes in *Chlorophyceae* were reconstructed with the online program RaxML 8.1.5 (Stamatakis 2006). The ML tree suggests that *Coelastrum* is closely related to *Pectinodesmus* and forms a sister-group with *Scenedesmus* (Figure 1). This result is in agreement with previous phylogenetic results constructed using the ITS sequence. However, additional mitochondrial data from *Scenedesmaceae* are needed to show a clear phylogenetic pattern.
